# Impact of Silibinin A on Bioenergetics in PC12APP_sw_ Cells and Mitochondrial Membrane Properties in Murine Brain Mitochondria

**DOI:** 10.3390/antiox10101520

**Published:** 2021-09-24

**Authors:** Carsten Esselun, Bastian Bruns, Stephanie Hagl, Rekha Grewal, Gunter P. Eckert

**Affiliations:** 1 Institute for Nutritional Sciences, Justus-Liebig-University of Giessen, 35392 Giessen, Germany; Carsten.Esselun@ernaehrung.uni-giessen.de (C.E.); Rekha.Grewal@ernaehrung.uni-giessen.de (R.G.); 2 Institute of Pharmacology, Goethe-University of Frankfurt am Main, 60439 Frankfurt am Main, Germany; bastian.bruns@gmx.net (B.B.); publications@s-hagl.de (S.H.)

**Keywords:** PC12APPsw, silybin, silibinin, flavolignan, mitochondria, NMRI, SNP, fluidity, swelling, aging

## Abstract

Age-related multifactorial diseases, such as the neurodegenerative Alzheimer’s disease (AD), still remain a challenge to today’s society. One mechanism associated with AD and aging in general is mitochondrial dysfunction (MD). Increasing MD is suggested to trigger other pathological processes commonly associated with neurodegenerative diseases. Silibinin A (SIL) is the main bioactive compound of the Silymarin extract from the Mediterranean plant *Silybum marianum* (L.) (GAERTN/Compositae). It is readily available as a herbal drug and well established in the treatment of liver diseases as a potent radical scavenger reducing lipid peroxidation and stabilize membrane properties. Recent data suggest that SIL might also act on neurological changes related to MD. PC12APP_sw_ cells produce low levels of human Aβ and thus act as a cellular model of early AD showing changed mitochondrial function. We investigated whether SIL could affect mitochondrial function by measuring ATP, MMP, as well as respiration, mitochondrial mass, cellular ROS and lactate/pyruvate concentrations. Furthermore, we investigated its effects on the mitochondrial membrane parameters of swelling and fluidity in mitochondria isolated from the brains of mice. In PC12APP_sw_ cells, SIL exhibits strong protective effects by rescuing MMP and ATP levels from SNP-induced mitochondrial damage and improving basal ATP levels. However, SIL did not affect mitochondrial respiration and mitochondrial content. SIL significantly reduced cellular ROS and pyruvate concentrations. Incubation of murine brain mitochondria with SIL significantly reduces Ca^2+^ induced swelling and improves membrane fluidity. Although OXPHOS activity was unaffected at this early stage of a developing mitochondrial dysfunction, SIL showed protective effects on MMP, ATP- after SNP-insult and ROS-levels in APPsw-transfected PC12 cells. Results from experiments with isolated mitochondria imply that positive effects possibly result from an interaction of SIL with mitochondrial membranes and/or its antioxidant activity. Thus, SIL might be a promising compound to improve cellular health when changes to mitochondrial function occur.

## 1. Introduction

Bioactive ingredients of plant extracts have been used in medicine for centuries. Silymarin, an extract from the Mediterranean plant *Silybum marianum* (L.) (GAERTN./Compositae), is a well-established none-prescriptive and readily available herbal drug in diseases affecting the liver, like non-alcoholic fatty liver disease, steatosis or non-alcoholic steatohepatitis [1,2,3]. Effects of Silymarin and its main bioactive ingredient Silibinin (SIL) have been attributed to their antioxidative properties scavenging reactive oxygen species (ROS) and reducing lipid peroxidation [4]. Furthermore, SIL has also been found to improve mitochondrial dysfunction in hepatic cell lines [1,5], which is also a hallmark of several hepatic diseases. Moreover, it has also been suggested that SIL could affect cholesterol levels in membranes, effectively modulating permeability and fluidity of hepatic membrane [6]. Additionally, effects on membrane permeability were also detected in mitochondria, as SIL was able to regulate Ca^2+^ influx, which is generally connected to the opening of the mitochondrial permeability transition pore (mPTP) [7,8].

Mitochondrial dysfunction (MD) is characterized by an overproduction of ROS and underperformance of the oxidative phosphorylation system (OXPHOS). MD is not only a prominent characteristic of neurodegenerative diseases including Alzheimer’s Disease (AD), but also a feature of aging. For a long time, the formation of hyperphosphorylated tau protein and of beta amyloid (Aβ) was the main focus of AD research. Due to the failure of clinical trials on drugs that target Aβ, recent studies have started to question the approach of lowering brain Aβ levels to treat AD [9,10]. Nowadays an approach targeting a much earlier stage in the progression of neurodegenerative diseases is recommended [11,12,13]. It is thought that prevention should start during physiological aging as it represents the most important risk factor for AD [12]. Age-related MD might trigger further pathological processes like Aβ accumulation and changes of tau protein phosphorylation [12,13]. Swerdlow et al. formulated the mitochondrial cascade hypothesis, which starts a vicious circle that finally results in pathological features of neurodegeneration [12,13].

MD is a key feature of aging [14,15,16] and is involved in several age-related diseases [17,18,19]. Therefore, enhancing mitochondrial function might be a promising way for the prevention and treatment of neurodegenerative diseases. Due to the slow onset of neurodegenerative diseases such as AD, being asymptomatic for decades, new strategies to improve aging must be found at the onset of the first modulations of mitochondrial function.

As SIL is already known to exhibit effects on hepatic mitochondria, it might also benefit neurodegenerative diseases associated with MD in the brain. Lee et al. reported that SIL stabilized mitochondrial membranes in an animal model for Parkinson’s Disease, effectively decreasing neuronal loss [20]. Furthermore, SIL attenuated oxidative stress in PC12 cells [21,22]. Liu et al. showed that SIL protected cells from nitrosative stress induced by the addition of sodium nitroprusside (SNP) [21]. Recently, we also reported that SNP-induced damages to ATP production and mitochondrial membrane potential (MMP) were effectively rescued by SIL in both PC12 and hepatic HepG2 cells [5]. There have also been studies showing that SIL could inhibit Aβ aggregation [23,24].

Here, we continue our initial research, which investigated the effects of SIL in neuronal and hepatic cells on mitochondrial function, in a cellular model of early AD [25], which is characterized by initial modulations to mitochondrial function. For this reason, we used PC12 cells transfected with the Swedish APP double mutation (APPsw) [26]. PC12 cells stem from the pheochromocytoma of the rat adrenal medulla and are a common model to investigate neurotoxicity/protection in undifferentiated cells [27,28]. The APPsw-transfection induces the production of low levels of human Aβ into the cell line and represents an established cellular model for molecular aspects of AD [29,30,31,32] and here, resulted in the onset of changes to mitochondrial function. 

This study further aimed to investigate SIL’s effect on the opening of the mitochondrial permeability transition pore (mPTP) and the membrane fluidity of mitochondria isolated from the brains of healthy NMRI mice based on earlier findings in neuronal cells [5]. 

## 2. Materials and Methods 

### 2.1. Chemicals

All chemicals used for this research were purchased from Merck (Darmstadt, Germany) in the highest purity available. Silibinin A (SIL) (purity 97%) was purchased from LKT Laboratories (St. Paul, MN, USA). It was solubilized in DMSO. Thus, DMSO was used as a control for all experiments in a concentration ranging from 1% to 0.1%, having no effect on the measured parameters. All aqueous solutions were prepared using type-1 ultrapure water. 

### 2.2. Animals

For all experiments on brain mitochondria, 6–7 months old, male NMRI mice were used. Mice were kept in the animal facility of the pharmacological institute of the Goethe-University Frankfurt am Main and received a standard maintenance diet, as they were not part of any experiments. All mice had ad libitum access to the diets and water. After sacrifice by decapitation, the brain was removed and separated from the cerebellum and olfactory bulb. All experiments were carried out by individuals with appropriate training and experience according to the requirements of the Federation of European Laboratory Animal Science Associations and the European Communities Council Directive (Directive 2010/63/EU).

### 2.3. Cell Lines

PC12neo [5,33] and PC12APP_sw_ were used as previously published [25,30,32,34]. PC12 cells were stably transfected with a pCMV vector and a neomycin resistance as control (PC12neo) or with the Swedish double mutation of human AβPP (PC12APP_sw_).

PC12 cells were cultivated in 250 mL Greiner flasks with Dulbecco’s Modified Eagle Medium (DMEM) (Gibco, Thermo Scientific, Waltham, MA, USA) supplemented with 10% (*v*/*v*) fetal bovine serum (FBS), 5% horse serum (HS) and 1% antibiotics (penicillin, streptomycin and G418). To maintain cell health and prevent overgrowth, cells were transferred to a new flasks when cell coverage reached 80–90% of the flask surface.

For experiments, cells were harvested, counted using a Neubauer Chamber and diluted to 10^6^ cells/mL. Cells were transferred into 24-well (MMP, 2 × 10^5^ cells/well) or 96-well plates (ATP/ROS, 2 × 10^4^ cells/well) in reduced DMEM (2% FBS, 1% HS). After 48 h cells were incubated with either SIL or DMSO as solvent control. To assess the effect of SIL on nitrosative stress, a subset of cells was incubated with 0.5 mM SNP 1 h after SIL exposure. After 24 h, cells were measured. 

### 2.4. Measurement of Mitochondrial Membrane Potential (MMP)

MMP was assessed using fluorescence dye rhodamine-123 (R123). Cells were incubated at 37 °C and 5% CO_2_ for 15 min with 0.4 µM R123. Cells were washed with Hank’s Balanced Salt Solution (HBSS) buffer (supplemented with Mg^2+^, Ca^2+^ and HEPES; pH 7.4; 37 °C) to remove excess fluorescence dye before being centrifuged at 750× *g* for 5 min. Medium was removed and cells were carefully resuspended in fresh HBSS. Fluorescence signal was measured at an excitation wavelength of 490 nm and the emission wavelength of 535 nm (Victor X3 2030 multilabel counter, Perkin Elmer, Waltham, MA, USA). The fluorescence was measured four times, normalized to the cell count and displayed relative to the control groups.

### 2.5. Measurement of ATP Concentrations

Bioluminescence kit ViaLight (Lonza, Basel, Switzerland), based on the reaction of ATP and luciferin, was used to determine ATP concentrations. Previously incubated cells were removed from the incubator and allowed to cool to room temperature for 10 min. Following incubation with lysis buffer for 10 min, a monitoring reagent was added to the cells for another 5 min. The emitted light was assessed with a luminometer (Victor X3 2030 multilabel counter, Perkin Elmer, Waltham, MA, USA). Concentration was determined via standard curve. Results were adjusted to cell count and displayed relative to the control group.

### 2.6. High-Resolution Respirometry

Respiration of cells and isolated mitochondria was measured using an Oxygraph-2k respirometer (Oroboros, Innsbruck, Austria) as described previously [35] using a protocol designed by Gnaiger et al. [36]. Respiration is shown in different states—(1) endogen: the endogenous respiration of cells; (2) Dig: the addition of 8 µM digitonin to disrupt cell membranes and remove naive substrates; (3) CI_(L)_: respiration after the addition of 10 mM glutamate and 2 mM malate to compensating for proton leaks through the membrane; (4) CI_(P)_: coupled complex I respiration after the addition of 2 mM ADP activating CI dependent ATP production; (5) CI&CII_(P)_: maximal coupled CI and CII respiration after the addition of 10 mM succinate; (6) CI&CII_(L)_: leak respiration of CI and CII after the addition of 2 µg/mL oligomycin to inhibit ATP synthase; (7) CI&CII_(U)_: maximal uncoupled CI and CII activity to compensate for increased proton transport into the matrix after the stepwise addition of Carbonyl cyanide p-trifluoromethoxyphenylhydrazone (FCCP) up to a total concentration of 0.5 µM; (8) CII_(U)_: uncoupled respiration using only CII substrates after CI inhibition via the addition of 0.5 µM rotenone; (9) CIV_(U)_: maximal uncoupled respiration of CIV after the addition of 2.5 µM antimycin A, which inhibits complex III, as well as the addition of the electron-donator 0.5 mM *N*,*N*,*N*′,*N*′-tetramethyl-p-phenylenediamine dihydrochloride (TMPD) and 2 mM of the TMPD-regenerating agent ascorbate. The residual oxygen consumption of enzymes not part of the oxidative phosphorylation was measured after the addition of antimycin A, and then subtracted from all stages of the experiment. Oxygen consumption after the addition of 12 mM NaN_3_ at the end of the experiment stemmed from the autoxidation of TMPD and was thus additionally substrated from CIV_(U)_. The respiratory control ratio, as an indicator for the state of coupling, was calculated from CI_(L)_ and CI_(P)_ data. Data were recorded using DatLab v. 4.3.2.7 was used. 

### 2.7. Citrate Synthase Activity

A subsample of cells from respiration experiments was immediately frozen and stored at −80 °C for the citrate synthases activity assay. Samples were thawed while the reaction medium (0.1 mM 5,5′-dithio-bis-(2-nitrobenzoic acid) (DTNB), 0.5 mM oxaloacetate, 50 µM EDTA, 0.31 mM acetyl coenzyme A, 5 mM triethanolamine hydrochloride and 0.1 M Tris-HCl) was mixed and heated to 30 °C for 5 min. Afterwards, a volume of 200 µL cell suspension was added to the reaction medium. Citrate synthases activity was determined at 412 nm as an increase in absorbance was linearly related to citrate synthases activity generating citrate from oxalacetate and acetyl-CoA. Each sample was measured in triplicates.

### 2.8. Protein Content

Protein content was determined using Pierce BCA Protein Assay Kit (Thermo Scientific, Waltham, MA USA) according to the manufacturer’s instructions of either freshly isolated mitochondria or thawed cells previously frozen from other experiments. Absorbance was measured using a ClarioStar plate reader (BMG Labtech, Ortenberg, Germany). 

### 2.9. Aβ_1-40_ Concentrations

Concentrations of Aβ_1-40_ were determined using Amyloid beta1-40 Kit (Cisbio, Perkin Elmer, Waltham, MA, USA) according to the manufacturer’s instructions. Cells were previously seeded in culture flasks until they reached a confluency of 70–80%, then incubated with 50 µM SIL or ctrl. Cells were harvested 24 h later, washed with PBS once and stored in PBS containing cOmplete^TM^, EDTA-free Protease Inhibitor Cocktail (Merck, Darmstadt, Germany) at −80 °C until experimentation. Upon thawing the samples, cells were lysed using Cell Extraction Buffer (Invitrogen, Thermo Scientific, Waltham, MA, USA) before applying the kit’s protocol. Fluorescence was measured using a ClarioStar plate reader with HTRF filters (BMG Labtech, Ortenberg, Germany) at an emission wavelength of 665 nm for the acceptor and 620 nm for the donor. Samples were measured in triplicates

### 2.10. ROS Concentrations

ROS levels were determined using fluorescence dye DCFDA in a Cellular ROS Assay Kit (abcam, Cambridge, UK) according to the manufacturer’s instructions. Fluorescence was measured using a ClarioStar plate reader (BMG Labtech, Ortenberg, Germany) with an excitation/emission wavelength of 485 nm/535 nm. Samples were measured in duplicates.

### 2.11. Lactate/Pyruvate Assay

Lactate and pyruvate concentrations were determined using Lactate Assay Kit and Pyruvate Assay Kit (Merck, Darmstadt, Germany) according to the manufacturer’s instructions. Cells grown in 250 mL Greiner flasks were incubated with 50 µM SIL or ctrl for 24 h, when they had reached a confluency of 70–80%. After 24 h cells were harvested from the flasks, washed once with PBS and stored at −80 °C. On the day of experimentation, samples were thawed and heated to 95 °C for 10 min, before the Assay kit was performed. Absorbance was measured using a ClarioStar plate reader (BMG Labtech, Ortenberg, Germany). Samples were measured in duplicates.

### 2.12. Isolation of Mouse Brain Mitochondria for Mitochondrial Membrane Interactions

Brains of mice were homogenized in Percoll-Isolation medium (IM). The cell suspension was applied to a Percoll gradient and centrifuged in three steps to separate brain mitochondria from the rest of brain tissue. Condition of mitochondria was determined by a short respirometry protocol and protein content was determined as earlier described, before mitochondrial membrane interactions were measured.

### 2.13. Mitochondrial Swelling (MS)

The activity of mitochondrial permeability transition pore (mPTP) was assessed by Ca^2+^ induced swelling of the mitochondria. The swelling of mitochondria was monitored photometrically by measuring the scattering of the light. 

In the beginning, 10 µL of healthy, isolated mitochondria was diluted in 1.1 mL measuring buffer (MB). After the addition of glutamate (5 mM) and malate (5 mM), the mixture was incubated for 8 min at 37 °C. The experiment was started with the addition of oligomycin (4.0 mg/mL). 

After 60 s, either 2 µL control, SIL or cyclosporin A was added to the mixture containing isolated mitochondria. After 1 min 4.4 µL ADP (20 µM) was added. Finally after 3 min into the experiment, the swelling was induced via the addition of 2 nmol/mg_Protein_ or 4 nmol/mg_Protein_ Ca^2+^. Cyclosporin A was chosen as an additional control substance since it is known to inhibit mitochondrial swelling by inhibiting the formation of the mPTP. The swelling was measured against maximum swelling attained by the addition of 5.5 µL alamethicin in the last stage of the experiment’s protocol. 

### 2.14. Mitochondrial Membrane Fluidity (MMF)

Mitochondrial membrane fluidity was analyzed as anisotropy calculated from the shift of linear polarized light. Trimethylamine-diphenylhexatriene (TMA-DPH) was used as fluorescence dye. The excitation wavelength was 354 nm and the detection wavelength was set to 450 nm. An amount of 30 µL of the intact mitochondria was diluted in 1070 µL of buffer, before the addition of 10 µL of SIL or ctrl. Mixtures were incubated at 37 °C for 30 min, before measurement. Lower anisotropy corresponded to high membrane fluidity. All samples were measured in 5-time repeats. 

### 2.15. Statistics

To calculate statistical significance, either a Student’s *t*-test, Mann–Whitney test or one-way ANOVA followed by Tukey’s post-hoc test was performed using GraphPad Prism version 8.0.1 for Windows (GraphPad Software, San Diego, CA, USA). Unless otherwise stated, data are presented as mean ± SEM. Statistical significance is displayed as follows: **** *p* < 0.0001; *** *p* < 0.001; ** *p* < 0.01; * *p* < 0.5. 

## 3. Results

**Figure 1:**
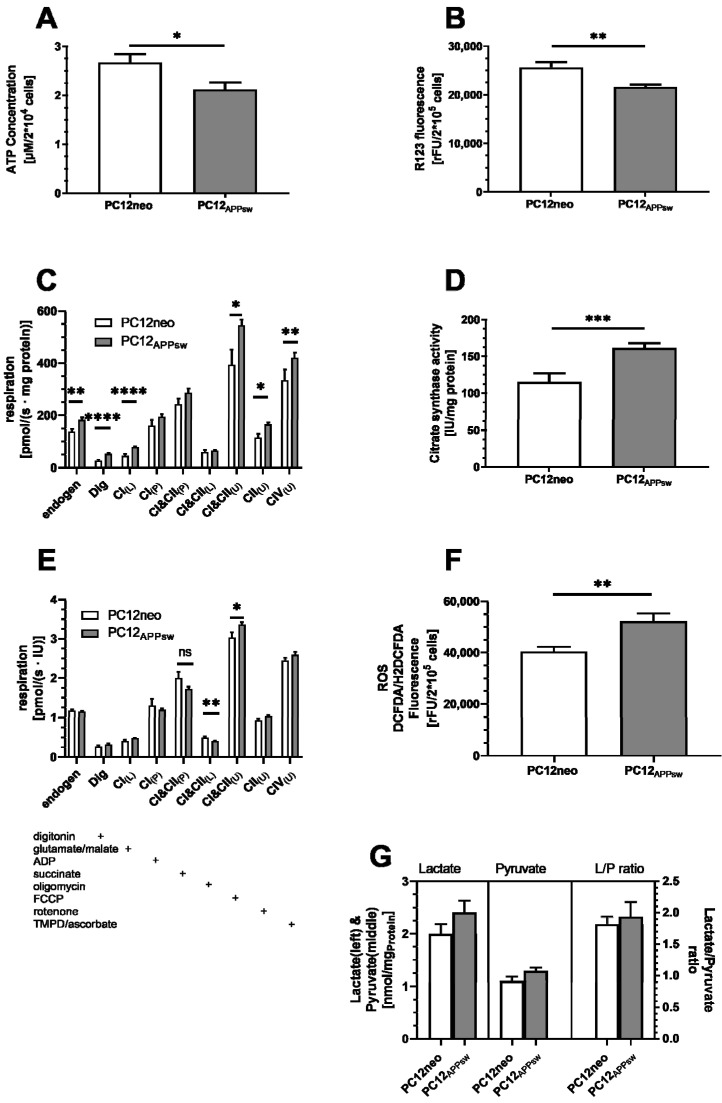
Comparison of mitochondrial and cellular parameters of PC12neo and PC12APP_sw_ cells. (**A**) Adenosine triphosphate (ATP) level of 2 × 10^4^ cells of both cell lines. (**B**) R123 fluorescence as a measure for mitochondrial membrane potential (MMP) level of 2 × 10^5^ cells of both cell lines. (**C**) Respiration adjusted to samples’ protein content. Activities of the oxidative phosphorylation (OXPHOS) complexes were measured by adding substrates, inhibitors or uncouplers for each complex. The substance added is indicated by a “+” marker under the graph. States denoted by either _(L)_, _(P)_ or _(U)_ describe a leaked state due to lack of ADP or inhibited ATP synthase, coupled, physiological respiration in the presence of substrates for the specific complex or uncoupled respiration following the addition of FCCP. (**D**) Citrate synthase activity adjusted to the protein content of the samples. (**E**) Respiration adjusted to Citratesynthase activity. (**F**) ROS Level measured as DCFDA/H2DCFDA fluorescence of 2 × 10^4^ cells. (**G**) Lactate and pyruvate concentration (left axis) adjusted to protein content and ratio of lactate/pyruvate (right axis). Data are displayed as mean ± SEM. *n* = 9–16. Statistical significance was tested via Student’s *t*-test (**** *p* < 0.0001, *** *p* < 0.001, ** *p* < 0.01, * *p* < 0.05, ^n.s.^ *p* > 0.05).

The comparison of PC12neo and PC12APP_sw_ cells, as models for healthy neuronal cells and cells affected by initial increases in amyloid-beta production, showed that the APPsw-transfection has a significant effect on mitochondrial function. Although, MMP (*p* = 0.003) and adenosine triphosphate (ATP) levels (*p* = 0.0236) are significantly decreased the complex activities of the OXPHOS system are significantly elevated in PC12APP_sw_ compared to PC12neo cells (Figure 1A–C). The respiration data show that endogenous oxygen consumption of PC12APP_sw_ cells is significantly increased compared to control cells (*p* = 0.0033). Additionally, complex activity of the respiratory chain appears to be generally increased in PC12APP_sw_ cells as there are trends in coupled states (CI_(P)_ *p* = 0.1699; CI+II_(P)_ *p* = 0.1079) or significant changes in uncoupled state (CI+II_(U)_ *p* = 0.0127; CII_(U)_ *p* = 0.0118; CIV_(U)_ *p* = 0.0026). Latter states, however, are artificially induced via the addition of FCCP and ascorbate/TMPD and do not occur in a physiological state of the mitochondria. These states provide information about the maximum possible activity of the complexes. The citrate synthases activity was significantly increased (Figure 1D, *p* = 0.001), which might at least in part explains the increased respiration shown in Figure 1C. 

If respiration is adjusted to citrate synthase activity, differences between both cell lines decrease and only CI&CII_(L)_ and CI&CII_(U)_ show significant differences. Since citrate synthase activity represents an established marker for mitochondrial mass [37], increased respiration may be a result of an increasing number of mitochondria. Still, ATP and MMP are decreased in PC12APP_sw_. Looking at the lactate and especially pyruvate concentration, both cell lines show no significant differences (Figure 1F) implying that although glycolysis playing a role in ATP production, this pathway appears to be similar in both cell lines. Furthermore, results for the lactate/pyruvate ratio suggest that the transfected cells do not compensate their changes to the ETC by increased glycolysis. Instead, our data imply that cells may try to counteract the reduced ATP and MMP by increased mitochondrial biogenesis. Still, although the mitochondrial mass is increased, reduced ATP and MMP might stem from modulations to the ETC. Table 1 shows the RCR of CI as an indicator for the state of coupling in both cell lines. The effect on coupling in PC12APP_sw_ was limited at best (*p* = 0.69), showing only a small trend for altered coupling, suggesting that OXPHOS was unaffected by the transfection. As the ETC is the major production site for ROS, we investigated cellular ROS levels and found significantly higher ROS levels (approx. 30%) in PC12APP_sw_ cells (Figure 1F, *p* < 0.0041).

**Figure 2:**
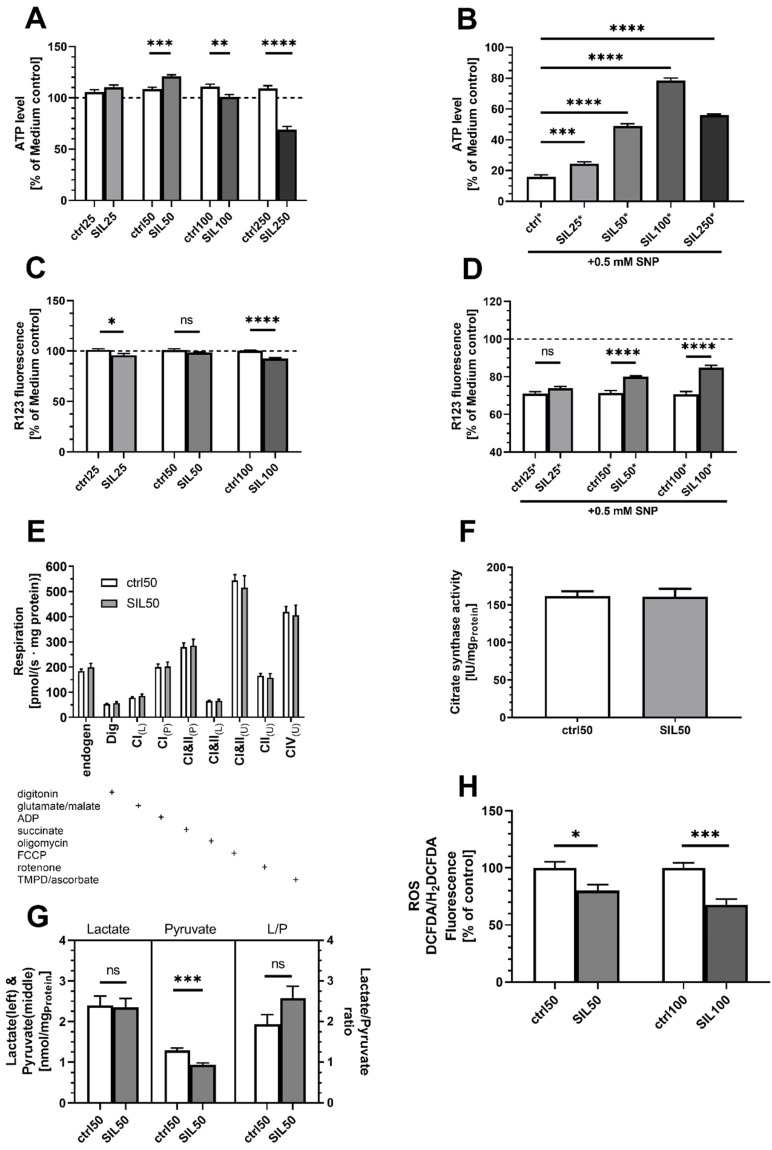
Mitochondrial function in PC12APP_sw_ cells treated with SIL in various concentrations. (**A**) ATP Level (% of medium control). Cells were incubated with SIL (SIL) in different concentrations (25–250 µM) for 24 h and measured against control cells treated with DMSO. DMSO treated cells showed no significant difference compared to the medium control. (**B**) ATP level (% of medium control) of cells injured via the addition of 0.5 mM SNP 1 h after the initial incubation with test substances. Cells were incubated for a total of 24 h. (**C**) MMP level (% of medium control). Cells were incubated with 25 µM–100 µM SIL for 24 h and measured against control cells treated with DMSO. DMSO treated cells showed no significant difference compared to medium control. (**D**) MMP level (% of medium control) of cells insulted via the addition of 0.5 mM SNP 1 h after the initial incubation with test substances. Cells were incubated for a total of 24 h. (**E**) Respiration of PC12APP_sw_ cells after 24 h of incubation with 50 µM SIL or solvent control. Activities of OXPHOS complexes were assessed by adding substrates, inhibitors or uncouplers specific to each complex. A “+” marker indicates which substance was added at which point of the experiment. States denoted by either _(L)_, _(P)_ or _(U)_ describe a leak state due to lack of ADP or inhibited ATP synthase, coupled, physiological respiration in the presence of substrates for the specific complex or uncoupled respiration following addition of FCCP. (**F**) Citrate synthases activity. Data are displayed as mean ± SEM. *n* = 8–16 Statistical significance was tested via one-way ANOVA and Tukey’s post-hoc test in (**B**). In (**A**,**C**–**H**), statistical significance was calculated via Student’s *t*-test of the treatment group versus the DMSO control (ctrl). Additionally, in (**H**), data for SIL50 did not fulfill the normality test according to Shapiro–Wilk, thus non-parametric Mann–Whitney test was performed to assess statistical significance. (**** *p* < 0.0001, *** *p* < 0.001, ** *p* < 0.01, * *p* < 0.5).

PC12APP_sw_ cells treated with 50 µM SIL showed a significant improvement in ATP levels compared to control cells (Figure 2A; *p* = 0.0002). While a lower concentration of 25 µM showed no effects, higher concentrations of 100 µM (*p* = 0.0073) and especially 250 µM (*p* < 0.0001) significantly reduced ATP levels compared to control (Figure 2A). The addition of SNP, to introduce a more severe stressor to mitochondrial function, inducing reactive nitrogen species (RNS) [21,38,39] and SIL was able to rescue the ATP (*p* < 0.0005 to 0.0001) production and MMP (*p* < 0.0001) across all tested concentrations (Figure 2B,D). Although 100 µM SIL shows the highest protective potential against SNP, only 50 µM SIL significantly enhanced basal ATP levels in PC12APP_sw_ cells (Figure 2A,B). This increase in ATP levels was neither due to an enhanced OXPHOS activity or MMP nor due to an enhanced mitochondrial mass since all parameters were unchanged (Figure 2C,E,F). Although PC12APP_sw_ did not show altered lactate or pyruvate levels compared to PC12neo, treatment of the PC12APP_sw_ cells led to a reduction in pyruvate (*p* = 0.0004), while lactate levels remained stable (Figure 2G). Figure 2H shows the SIL’s effect on cellular ROS: A concentration of 50 µM was able to reduce ROS by around 20% (*p* = 0.035) while 100 µM lowered ROS by 33% (*p* = 0.0002), rescuing PC12APP_sw_ cell to the same level as PC12neo cells. To evaluate if the effects of SIL reported herein are a consequence of possible inhibition of Aβ expression, we investigated SIL’s effects on Aβ_1-40_ production. It turned out that incubation of PC12APP_sw_-cells with SIL did not reduce Aβ_1-40_ levels in our cellular model which is based on Aβ overexpression (Table 2). 

**Figure 3:**
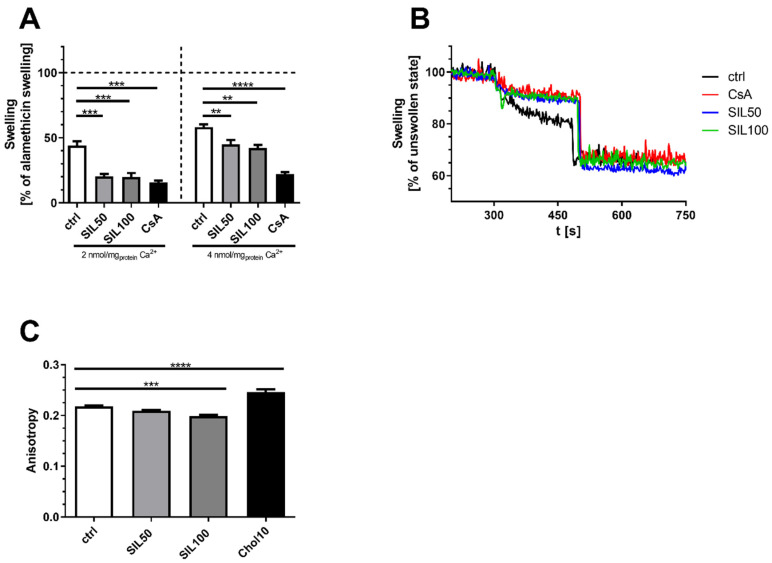
Mitochondrial swelling and membrane fluidity in freshly isolated mitochondria of aged NMRI mice. (**A**) Mitochondrial swelling (% of maximum alamethicin swelling). Freshly isolated mitochondria were activated by the addition of 5 mM glutamate, 5 mM malate and 4 mg/mL oligomycin. Mitochondria were treated with 50 µM or 100 µM SIL (SIL), control (ctrl) or Cyclosporin A as negative control and an additional 20 µM ADP. The swelling was induced via the addition of 2 nmol/mg_Protein_ Ca^2+^ or in an identical setup with 4 nmol/mg_Protein_ Ca^2+^. Protein content of samples was determined via the BCA method. The addition of 8.5 µg/mL alamethicin at the end resulted in maximum swelling of mitochondria. (**B**) Visual representation of an exemplary experiment with 2 nmol/mg_Protein_ Ca^2+^. Calcium was added after 300 s and alamethicin after 500 s. (**C**) Mitochondrial membrane fluidity measured as anisotropy in intact brain mitochondria from aged NMRI mice. Anisotropy was measured using TMA-DPH as marker. Mitochondria were incubated with SIL (SIL) in different concentrations (50 µM and 100 µM) and measured against control cells treated with DMSO (ctrl). As positive control, 10 µM Cholesterol (Chol10) was used as it is known to reduce membrane fluidity. Displayed are means ± SEM. *n* = 8–10. Statistical significance was tested in all experiments via one-way ANOVA and Tukey’s post-hoc test. Statistical analysis in A was determined for each Ca^2+^ concentration separately. (**** *p* < 0.0001, *** *p* < 0.001, ** *p* < 0.01).

The introduction of radicals like RNS or ROS leads to damage of mitochondria and peroxidation of lipids in the membranes. This in return induces more ROS/RNS [40,41,42] but can also affect calcium homeostasis which is involved in mitochondrial swelling, rupture and cytochrome C release into the cytosol ultimately leading to apoptosis [43,44]. Since we found reduced ROS levels, investigation of SIL’s effect on the opening of the mitochondrial permeability transition pore (mPTP) was the next step. For this, freshly isolated mitochondria from the brain of NMRI mice were treated in vitro with 50 µM or 100 µM SIL. Results showed that 50 µM (*p* < 0.0001) and 100 µM SIL (*p* < 0.0001) significantly reduced Ca^2+^ induced swelling of mitochondria, as seen in Figure 3A,B. Moreover, if the swelling was induced with 2 nmol/mg_Protein_ Ca^2+^, effects observed for SIL were similar to Cyclophilin D-binding Cyclosporin A, which reduces the probability for mPTP opening. Even if the Ca^2+^ concentration was doubled to increase the pressure of H_2_O influx into the mitochondria, SIL still significantly attenuated (*p* < 0.0031; *p* < 0.0011) membrane swelling in both concentrations. Inhibition of mitochondrial rupture through swelling, might be one of the ways SIL is able to attenuate mitochondrial dysfunction resulting in reduced ROS production in the OXPHOS system and restored ATP production. 

Next, mitochondrial membrane fluidity was assessed in freshly isolated mitochondria from NMRI mice (Figure 3C) to investigate if SIL interacts with mitochondrial membranes. Using TMA-DPH, a fluorescent dye that imbeds itself into outer, hydrophilic regions of the phospholipid layer, we found that 50 µM SIL showed a trend and 100 µM SIL significantly increased membrane fluidity (*p* < 0.0004) which is inversely correlated to the measured anisotropy. Cholesterol (Chol) stabilizes membranes and was used as a control (Figure 3).

## 4. Discussion

Here, we investigated the mitochondrial function of PC12APP_sw_ cells, a model for early AD characterized by Aβ production [26,45] and early changes to mitochondrial function as well as PC12neo cells containing an empty vector as control. Furthermore, we used SNP to induce nitrosative stress to enhance mitochondrial dysfunction a common hallmark of AD [46,47,48]. 

Comparing PC12neo with PC12APP_sw_ cells, we found significantly lower mitochondrial membrane potential and production of ATP. Activities of the OXPHOS complexes on the other hand were generally increased. The finding that the citrate synthase activity, a well-established marker for mitochondrial content [37], was significantly elevated might indicate enhanced mitochondrial biogenesis in PC12APP_sw_ cells, potentially compensating for the deficient energy production. The involvement of mitochondrial biogenesis is likely as respiration of cells adjusted to mitochondrial mass is very similar in both cell lines. Differences found in leak respiration could result from damages to membranes induced by, e.g., ROS, which was significantly elevated in PC12APP_sw_ cells. Significantly increased uncoupled respiration has to be considered carefully, as the uncoupled state is an artificial state indicating the maximal activity of the complexes. Here, it might be another result of a higher mitochondrial mass. Mitochondria are not capable to repair naturally occurring mutations and rely on processes like fission and fusion to maintain cellular function or are degraded in a process called mitophagy [49,50]. Taking this into account, since the mitochondrial mass was increased, decreased ATP and MMP levels could also suggest an accumulation of dysfunctional mitochondria providing less efficient energy production and increased generation of ROS [49]. This is also underlined by significantly increased ROS levels in PC12APP_sw_ cells compared to PC12neo and is generally in line with other studies. Butterfield et al. or Yatin et al. reported that increased Aβ production leads to increased LPO [51], ROS [52], and MD [53]. Nevertheless, the data for ROS must be interpreted with caution because DCFDA oxidation is also affected by compounds such as cytochrome C and heme oxidases [54]. Therefore, increased mitochondrial number, could have artificially elevated ROS levels in this comparison. Taken together, the effect of the APPsw transfection on mitochondrial function was rather limited. For this reason, ATP and MMP experiments with SIL were also conducted under additional stress applying SNP to induce an increased level of reactive compounds.

Recently, there have been reports that found SIL to be highly protective against nitrosative stress induced via SNP in PC12 cells [5,21]. Nitrosative/oxidative stress is commonly occurring during aging and neurodegenerative diseases and is mainly resulting from mitochondrial dysfunction and overproduction of ROS/RNS in the OXPHOS system [46,55]. Especially in the case of AD, the most common form of dementia, recent studies have started to question whether the amyloid beta cascade hypothesis is actually a reasonable target in search of a cure for the disease [9,10,56,57]. Swerdlow et al. proposed a different hypothesis for the onset of AD starting with increasing mitochondrial dysfunction during the process of aging resulting in a vicious circle that does not only lead to the well-documented accumulation of Aβ and hyperphosphorylated tau protein, but in return also aggravates MD [12,13]. Since citrate synthase activity was virtually identical in both groups, thus mitochondrial number unaltered, DCFDA fluorescence is easier to interpret. Our results suggest a protective effect of SIL against nitrosative stress and cellular ROS. High SIL concentrations (250 µM) retain their protective properties against SNP although they already exhibit toxic effects on basal MMP and ATP-levels. This is in agreement with Matsuo et al. and summarized by Procházková et al. who found that high concentrations of flavonoids can induce toxic levels of ROS [58,59]. This is also supported by our recent findings showing a significant reduction in MMP and ATP-levels in both PC12 as well as hepatic HepG2 cells [5]. Of all concentrations tested, 100 µM appeared to be the most protective. Yet, this concentration proved to be slightly toxic on basal levels of MMP and ATP. For this reason, we chose 50 µM SIL for the further experiments, as it not only improved basal ATP but also significantly retained ATP levels when cells were treated with SNP. However, OXPHOS and citrate synthase activity was unaffected after the treatment with SIL. A study by Dorta et al. found that respiration was generally unaffected by several flavonoids in a concentration of 50 µM [60]. SIL’s effect on ATP production not being linked to alterations to the mitochondrial respiratory chain complexes is also in agreement with our observations in PC12neo and HepG2 cells [5]. Besides oxidative phosphorylation that produces the majority of cellular ATP, it is also produced during glycolysis. Our data, however, suggest that SIL-treated cells have lower levels of pyruvate although lactate concentrations remain stable. This suggests that energy production is shifted away from glycolysis and OXPHOS and more towards anaerobic metabolism. Similarly, in perfused rat hepatocytes, SIL dose-dependently reduced glycolysis and had a dramatic effect on oxidative phosphorylation [61]. Still, it has to be mentioned that mitochondria in PC12APPsw cells could not be considered dysfunctional, as data suggested relatively little effect on mitochondrial function. Therefore, results in APPsw-transfected cells are similar to those obtained in SIL-treated PC12neo cells [5]. 

Looking at amyloid-beta levels itself, it turned out that incubation of PC12APP_sw_ cells with SIL for 24 h did not reduce Aβ levels. To the best of our knowledge, this is the first report of the effects of SIL on Aβ levels in PC12APP_sw_ cells. One conclusion of this experiment is that the effects that we report on mitochondrial function, oxidative stress, and glycolysis are independent of the expression of Aβ. Recently, we reported similar effects for the olive polyphenol ligstroside. Nanomolar concentrations of this secoiridoid improved mitochondrial dysfunction in SY5Y-APP695 cells, another Aβ-overexpression model [62]. Meng et al. who insulted PC12 cells, pre-treated with a mixture of curcumin, vorinostat and 1 µM SIL, with Aβ_25-35_ for 24 h, showed that this mixture could attenuate Aβ-induced apoptosis, oxidative stress and improve cell viability in general [57]. However, the authors did not report whether SIL had an effect on Aβ levels. Although we demonstrate that SIL does not inhibit Aβ expression it is possible that SIL affected Aβ aggregation. Yin et al. and Duan et al. provided evidence for SIL’s property to inhibit the aggregation of Aβ in an APP/PS1 mice model of AD [22] as well as in neuroblastoma SH-SY5Y cells [23]. Yin et al. found that SIL concentrations between 10 µM and 100 µM SIL inhibited Aβ aggregation by around 50% to 70% after an incubation time of 72 h [23]. 

Taken together, SIL did not affect the mitochondrial function or Aβ levels itself. Still, we were able to demonstrate a strong antioxidative ability in these APPsw-transfected cells. Although it is difficult to come to a conclusion about SIL’s effect on dysfunctional mitochondria, future studies should thoroughly address, the results suggesting that SIL’s properties are solely based on its qualities as an antioxidant. 

Results for swelling and membrane fluidity of mitochondria suggest that SIL’s effect might be related to membrane changes. Increased ROS production leads to changes of calcium homeostasis [63,64], i.e., by the opening of the mitochondrial permeability transition pore, leading in return not only to swelling but also to further increases in ROS. Zorov et al., who reported this, described this cycle as ROS-induced ROS Release (RIRR) [41,42]. Increasing uptake of Ca^2+^ through the mitochondrial calcium uniporter (MCU) leads to opening of the mPTP and influx of water which results in the swelling of mitochondria and potentially rupture of the outer membrane. These mitochondria not only lose their function, but also release pro-apoptotic substrates into the cytosol finally leading to the death of the cell [63]. Similar to cyclosporin A which inhibits the cyclophilin D-mediated mPTP opening, SIL also significantly reduces swelling resulting from exposure of cells to high levels of Ca^2+^. Since ROS is an inducer of mPTP opening, these results could be attributed to SIL’s antioxidative properties reducing ROS, as shown for PC12 cells. Another explanation would be that SIL directly affects mitochondrial membranes. This is in agreement with multiple studies coming to similar conclusions for SIL and Silymarin [65,66,67]. Yet, a more recent study showed that Silymarin extract’s effect on membrane stabilization was superior to that of SIL alone [68]. Our results of the experiments on membrane fluidity are also in line with the findings from Farghali et al. in perfused rat hepatocytes after Silymarin treatment [69]. Yao et al. also found a similar increase in membrane fluidity in liver cells following SIL treatment of rats adhering to a high-fat diet [70]. Similar to our results using TMA-DPH to monitor hydrophilic regions of the bilipid layer, Parasassi et al. additionally found that SIL also improved membrane fluidity in hydrophobic regions of microsomal membranes following SIL treatment [71]. 

Although Silibinin provides strong antioxidative and membrane stabilizing properties, its poor bioavailability could be a concern. Different formulations have been developed and are in use with the purpose to increase intestinal uptake. Pérez-Sánchez et al. evaluated the intestinal permeability of the most common forms of SIL, Siliphos—a Silibinin-phosphatidylcholine complex [72], and Euromed—a patented milk thistle extract used in commercially available Legalon [73], in an in vitro trans-well system using Caco-2 cells [74]. Based on the penetration coefficients, the tested SIL formulation was characterized as a moderately absorbed group of compounds and only Euromed could be considered a good candidate to cross the blood–brain-barrier (BBB) [74]. However, several in vivo studies used non-formulated SIL to investigate its effects on the brain [23,75,76,77,78,79,80]. Although brain levels were not reported in those studies, one could conclude that SIL or one of its metabolites would have been crossed the BBB to provide effects in the central nervous system.

## 5. Conclusions

In conclusion, our results show that the mitochondrial function of PC12APP_sw_ cells was comparatively little changed compared to control cells. Thus, the use of this model has to be considered with caution and future studies should investigate SIL’s effect on mitochondrial function in more severely impaired models. Nevertheless, SIL led to increased ATP levels, lowered ROS levels and showed strong antioxidative potential against SNP-induced RNS in this cellular model of early AD. In isolated murine brain mitochondria, SIL is able to improve membrane fluidity, as well as to decrease Ca^2+^ induced mitochondrial swelling. Taken together, SIL showed strong antioxidative abilities in APPsw-transfected PC12 cells. 

## Figures and Tables

**Table 1:** Respiration control ratio of PC12neo and PC12APP_sw_ cells. RCR was calculated from CI_(L)_ and CI_(P)_ acquired in the respiration experiment. *n* = 11. Displayed are the means ± SEM. Statistical significance was tested via Mann–Whitney *t*-test (*p*^n.s.^ > 0.05).

Cells	State 3 Respiration (CI_(P)_)	State 4 Respiration (CI_(L)_)	RCR
PC12neo	1.30 ± 0.17	0.407 ± 0.036	3.11 ± 0.60
PC12APP_sw_	1.19 ± 0.040	0.478 ± 0.014	2.52 ± 0.14 *p*^n.s.^ = 0.69

**Table 2:** Aβ_1-40_ levels in PC12APP_sw_ cells treated with 50 µM SIL. Aβ was determined via Homogeneous Time-Resolved Fluorescence (HTRF). Data are adjusted to protein content of the samples. Data are displayed as mean ± SEM. *n* = 10. Statistical significance was tested via Student’s *t*-test (^n.s.^ *p* > 0.05).

	Ctrl	SIL50	*p*
Aβ_1-40_ [pg/mg_Protein_]	82.22 ± 7.98	99.26 ± 10.08	0.20, n.s.

## Data Availability

Data is contained within the article.
